# Ginkgo biloba extract EGb761 attenuates brain death-induced renal injury by inhibiting pro-inflammatory cytokines and the SAPK and JAK-STAT signalings

**DOI:** 10.1038/srep45192

**Published:** 2017-03-23

**Authors:** Yifu Li, Yunyi Xiong, Huanxi Zhang, Jun Li, Dong Wang, Wenfang Chen, Xiaopeng Yuan, Qiao Su, Wenwen Li, Huiting Huang, Zirong Bi, Longshan Liu, Changxi Wang

**Affiliations:** 1 Organ Transplant Center, The First Affiliated Hospital of Sun Yat-sen University, Guangzhou 510080, People’s Republic of China; 2 Organ Transplant Center, The First Affiliated Hospital of Wenzhou Medical University, Wenzhou 325000, People’s Republic of China; 3 Department of Clinical Laboratory, The First Affiliated Hospital of Sun Yat-sen University, Guangzhou 510080, People’s Republic of China; 4 Department of Pathology, The First Affiliated Hospital of Sun Yat-sen University, Guangzhou 510080, People’s Republic of China; 5 Laboratory Animal Center, The First Affiliated Hospital of Sun Yat-sen University, Guangzhou 510080, People’s Republic of China; 6 Guangdong Provincial Key Laboratory of Organ Donation and Transplant Immunology, Guangzhou 510080, People’s Republic of China; 7 Institution of Organ Donation and Transplant Immunology, Sun Yat-sen University, Guangzhou 510080, People’s Republic of China

## Abstract

This study aimed to investigate the protective effects of EGb761, a *Ginkgo Biloba* extract, against brain death-induced kidney injury. Sixty male Sprague Dawley rats were randomly divided into six groups: sham, brain-death (BD), BD + EGb b48h (48 hours before BD), BD + EGb 2 h (2 hours after BD), BD + EGb 1 h, and BD + EGb 0.5 h. Six hours after BD, serum sample and kidney tissues were collected for analyses. The levels of blood urea nitrogen (BUN) and serum creatinine significantly elevated in the BD group than in sham group. In all the EGb761-treated BD animals except for the BD + Gb 2 h group, the levels of BUN and serum creatinine significantly reduced (all *P* < 0.01). EGb761 attenuated tubular injury and lowered the histological score. In addition, the longer duration of drug treatment was, the better protective efficacy could be observed. EGb761 significantly reduced IL-1β, IL-6, TNF-α, MCP-1, IP-10 mRNA expression and macrophage infiltration in the kidney. EGb761 treatment at 48 hour before brain death significantly attenuate the levels of p-JNK-MAPK, p-p38-MAPK, and p-STAT3 proteins (all P < 0.05, compared to BD group). In summary, our data showed that EGb761 treatment protected donor kidney from BD-induced damages by blocking SAPK and JAK-STAT signalings. Early administration of EGb761 can provide better protective efficacy.

Kidney transplantation is current most optimal therapy for patients with irreversible chronic kidney failure, which can significantly prolong survival and improve quality of life of patients^1^. However, organ transplantation is severely limited by the donor availability. Since the Harvard criteria for brain death has been introduced in 1968 2, donation after brain death (DBD) has been being routinely performed in many countries. Although donation after cardiac death has been gradually increasing recently, DBD remains the main source of donor grafts in many countries at present^3^. It is generally accepted that the quality of grafts plays a crucial role in the transplantation outcome^4^. Particularly, compared to living-donor kidney transplantations, patients received kidneys from deceased donors have been shown to be of a significantly lower long-term patients and graft survival rate^5^,^6^. Brain death usually results from massive acute cerebral injury, induces systemic vasoconstriction and hypoperfusion of multiple organs, and eventually causes ischemia-hyperoxia injury in the donor kidneys^7^,^8^. The pathophysiological alterations also triggers a cascade of hemodynamic, inflammatory, and immunologic events^9^,^10^,^11^. Therefore, developing strategies for protection of BD-induced kidney injury are urgently required, which would help to improve the graft quality and the outcome of graft transplantation.

Immunohistochemical study of kidney biopsies have found that the elevated level of neutrophil infiltration and P-selectin expression were detected after reperfusion in deceased donor kidney grafts, which was associated with long cold ischemia times and delayed graft function, indicating that early inflammatory events account for the poor outcome of kidneys derived from deceased donors^12^. Hence, ischemic and inflammatory injuries are considered to be the two main contributors for brain death-induced kidney injury. Accordingly, a variety of strategies aiming to overcome these two targets have been developed to prevent brain death-induced kidney injury. For instance, cardiovascular support strategies including pretreatment of dopamine^13^ and catecholamine^14^, as well as anti-inflammatory strategies including pretreatment of recombinant soluble P-selectin glycoprotein ligand^15^, steroid^16^, carbamylated erythropoietin^17^, sodium nitrite^18^ and induction of heme oxygenase 1^19^ have been adopted to improve the quality of graft.

*Ginkgo biloba* is a kind of medicinal herb used in traditional Chinese medicine for several thousand years. EGb761 is a compound extracted from *Ginko biloba* leafs that consists of two active components; a flavonoid glycoside and terpene lactone^20^. The toxicological and pharmacological properties of EGb761 are well*-*characterized. A number of preclinical studies have suggested neuroprotective effects for EGb761^21^,^22^, and it have been widely used in prevention and treatment of Alzheimer’s disease and dementia in elder individuals^23^. The molecular mechanisms of neuroprotective effects of EGb761 are of multiple-aspects, including anti-apoptosis, anti-inflammatory, protecting mitochondria^24^, etc. Accumulating evidences also demonstrate that EGb761 possesses good protective effect on cardiovascular diseases^25^,^26^,^27^. Recent study showed that EGb761 exerts its anti-inflammatory effect by inhibiting several pro-inflammatory mediators and signaling pathways^28^. In addition, EGb761 has been shown to be capable of reducing ischemia-reperfusion (I/R)-induced renal injury in rats model^29^. Song *et al*. have also demonstrated that EGb761 possesses the protective effect against cisplatin-induced renal injury in rats model^30^.

All the above findings suggest that EGb761 may have potential beneficial effects against brain death-induced kidney injury. However, the feasibility of application of EGb761 on pharmacologic donor management is still unknown. In addition, the molecular mechanisms underlying the protective effect of EGb761 are still not fully understood. Therefore, the purpose of this study was to evaluate the protective effect of EGb761 against brain-death induced kidney injury and the underlying molecular mechanism of the protective effect. Adopting a rat model of brain death, the protective effect of EGb761 was demonstrated. We also demonstrated evidences supporting that JAK-STAT and SAPK signalings were involved in the protective effect of EGb761.

## Materials and Methods

### Animals

Sixty male Sprague Dawley (SD) rats aging 16–20 weeks and weighing 350–400 g were randomly assigned into six groups; the sham operated group (sham), the brain death group (BD), BD + EGb b48h (injected with EGb761 at 48 hours before BD), BD + EGb 2 h (injected with EGb761 at 2 hours after BD), BD + EGb 1 h, and BD + EGb 0.5 h. This study was approved by the Institutional Animal Care and Use Committee (IACUC) of the First Affiliated Hospital of Sun Yat-sen University and all experiments were carried out in accordance with relevant guidelines, including any relevant details.

### Brain death model of rat and EGb761 treatment

Animals were anesthetized intraperitoneally with chloral hydrate and then the left femoral artery was cannulated. The cannula was connected to a pressure transducer and used to continuously monitor blood pressure. A tracheotomy was performed, followed by intratracheal intubation. A hole was made in the skull at the left frontal bone 0.3 cm from the sagittal line. A Fogarty tube was inserted outside of the dura mater, and the tip was pointed in the caudal direction. The tube was stabilized for 30 min, and normal saline was injected into the balloon with a micro-infusion pump (RWD252, RWD Life Science Co., Ltd, Shenzhen, China) at a speed of 40 μL/min until breathing stopped. Mechanical ventilation was immediately initiated at a respiratory rate of 100/min and tidal volume of 2.0 mL with a small animal ventilator (HX-100E, Taimeng Software Co. LTD, Chengdu, China). Respiratory arrest and the disappearance of the corneal reflex and pupillary light reflex were used to determine brain death. Mechanical ventilation was provided for another 6 h after brain death. When the mean arterial pressure was reduced to 80 mmHg, normal saline was retracted to reduce the balloon until blood pressure recovered. The body temperature was maintained at 38.0 °C. In the sham group, a hole was made at the skull without insertion of a balloon tube and mechanical ventilation was provided for 6 h after the hole was made.

The animals treated with EGb761 received 50 mg/kg injected into the penile vein at 48 hours before surgery to induce brain death and 0.5, 1 and 2 hours after brain death, respectively. In the BD and sham groups, an equivalent volume of normal saline was injected at the same time points.

### Specimen Collection

After 6 h of mechanical ventilation, the abdominal aorta was punctured, and the blood was collected into a procoagulant inert gel tube. The inferior vena cava was opened, and normal saline (30 mL) was injected from the cephalic end of the aorta to flush both kidneys. The left kidney was removed, and its cortex was harvested. A fraction of the cortex was stored at 4 °C in 2.5% glutaraldehyde–2% paraformaldehyde in 0.1 mol/L PBS, and the remaining cortex was snap frozen in liquid nitrogen. The right kidney was divided along the sagittal plane and fixed in 4% paraformaldehyde.

### Serum creatinine (Cr) and blood urea nitrogen (BUN) assay

Blood sample was centrifuged at 1000 g for 10 min, and the supernatant was harvested. Serum Cr and BUN was measured by dry chemistry with an automatic biochemical analyzer (7180, Hitachi, Ibaraki-ken, Japan).

### Histopathological and immunohistochemical (IHC) assessment

Paraformaldehyde-fixed kidney was used to prepare paraffin-embedded tissue sample. The paraffin-embedded tissue sections (5-μm thick) were stained with hematoxylin-eosin (H&E) or immunochemistry staining (IHC) (anti-CD68 mAb, Cat. ab31630, Abcam, USA), and observed under a light microscope (Eclipse 80i, Nikon, Tokyo, Japan). For histopathological assessment, representative images were captured with a camera (DS-U1, Nikon, Tokyo, Japan) and analyzed with the ACT-2U image analysis software (version: 1.40.85.221, Nikon, Tokyo, Japan). Tubular injury was evaluated independently by two pathologists blinded to the treatments. Ten fields were randomly selected at a magnification of 400× and 10 tubules were selected from within each field. The scoring was performed as previously described^31^. Briefly, slides were evaluated based on the presence and extent of tubular epithelial cell flattening (1 point), brush border loss or shedding (1 or 2 points), the presence of a cast (2 points), and caducous necrotic cells without formation of cast or cell debris (1 point). For IHC assessment, ten fields were randomly selected from the slide of each animal at a magnification of 400×, and the number of positively stained cells was counted. Six rats were evaluated for IHC staining in each group.

### Transmission electron microscopy

Glutaraldehyde-fixed tissues were fixed in osmic acid, dehydrated in ethanol, and embedded. The tissue was cut into 0.05-μm sections and stained with uranyl acetate and lead citrate. These sections were observed under a transmission electron microscope (Tecnai G^2^ Spirit Twin, FEI, Brno, Czech Republic), and representative images were captured.

### Real-time RT-PCR

Total RNA was extracted from kidney using E.Z.N.A.^TM^ Total RNA Kit II (R6931-01, Omega Bio-tek, Norcross, GA, USA) according to manufacturer’s protocol. Reverse transcription was performed using the PrimeScript RT Master Mix (TakaRa, Dalian, China). Real-time PCR was performed using SYBR Premix Ex Taq^TM^II reagent (TakaRa) in a real-time quantitative thermal cycler (7900HT, Applied Biosystems, Foster City, CA, USA) with the following cycle conditons: an initial denaturation step (95 °C for 30 s), followed by 40 cycles of denaturation (95 °C for 5 s) and annealing-extension (60 °C for 30 s), with a final melt-curve stage (95 °C for 15 s, 60 °C for 60 s, and 95 °C for 15 s). GAPDH served as an internal reference, and data were analyzed using the 2^−ΔΔCt^ method. The primers for the target genes were: IL-1β, CCCTGAACTCAACTGTGAAATAGCA and CCCAAGTCAAGGGCTTGGAA; IL-6, ATTGTATGAACAGCGATGATGCAC and CCAGGTAGAAACGGAACTCCAGA; TNF-α, TCAGTTCCATGGCCCAGAC and GTTGTCTTTGAGATCCATGCCATT; monocyte chemoattractant protein-1 (MCP-1), CTATGCAGGTCTCTGTCACGCTTC and CAGCCGACTCATTGGGATCA; and CXCL10 (IP-10), TTATTGAAAGCGGTGAGCCAAAG and GCTGTCCATCGGTCTCAGCA. The primers for GAPDH were GGCACAGTCAAGGCTGAGAATG and ATGGTGGTGAAGACGCCAGTA.

### Western blot analysis

Total protein was extracted from the kidney tissues snap frozen in liquid nitrogen using RIPA lysis buffer [50 mM Tris (Ph 7.4), 150 mM NaCl, 1% Triton X-100, 1% sodium deoxycholate, 0.1% SDS, sodium orthovanadate, sodium fluoride, EDTA, and leupeptin] containing PMSF and phosphatase inhibitor (Forevergen, Guangzhou, China). The lysates were separated using polyacrylamide gel electrophoresis and transferred onto a PVDF membrane (Millipore, Billerica, MA, USA). The membranes were blocked with blocking buffer (Beyotime, Haimen, China) at room temperature and then incubated with one of the following primary antibodies:

Signal transducer and activator of transcription 3 (STAT3) (D3Z2G) (Cat. 12640, CST, USA), Phospho-STAT3 (Tyr705) (Cat. 9145), p38 mitogen-activated protein kinase (MAPK) (D13E1) (Cat. 8690), phospho-p38 MAPK (Thr180/Tyr182) (Cat. 9215), SAPK/JNK (Cat. 9252), phospho-SAPK/JNK (Thr183/Tyr185) (Cat. 4671). The membranes where then washed with TBST and then incubated with a horseradish peroxidase-affinity-purified Goat Anti-Rabbit IgG (H + L) secondary antibody (Jackson ImmunoResearch Laboratories, Inc., West Grove, PA, USA) for 1–2 h. Visualization was performed using chemiluminescence (Pluslight ECL Kit, Forevergen, Guangzhou, China). The representative images were captured and analyzed with the Quantity one software (Bio-Rad, Hercules, CA, USA). The optical density of the bands was determined. The expression of the target proteins was normalized to an internal control, GAPDH (D16H11) (Cat. 8884). All the antibodies were from Cell Signaling Technology, Boston, MA, USA.

### Statistical analysis

Statistical analysis was performed with SPSS version 13.0 for Windows (Chicago, Illinois, USA). Data were expressed as mean ± standard deviation. A one-way analysis of variance was used to compare among groups. When a significant difference was observed, post-hoc comparisons between two groups were performed. When variance was homogenous, the LSD test was used to compare between two groups. When variance was heterogeneous, the Dunnett’s T3 test was employed to compare between two groups. A value of *P* < 0.05 was considered statistically significant.

## Results

### EGb761 attenuated brain death-induced renal injury

**Figure 1:**
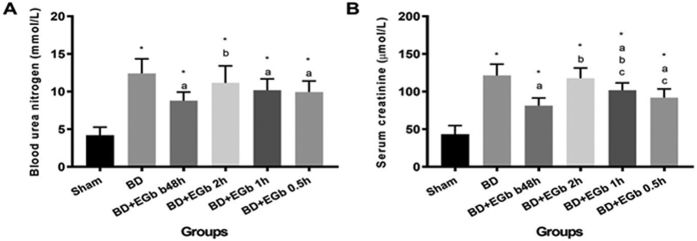
EGb761 treatment reduced serum creatinine (Cr) and blood urea nitrogen (BUN) level in brain death rats. Data are presented as means ± SD. ^*^P < 0.01 *vs*. sham, ^a^*P* < 0.01 *vs*. BD, ^b^*P* < 0.01 *vs*. BD + EGb b48h, ^c^*P* < 0.01 *vs*. BD + EGb 2 h, ^d^*P* < 0.01 *vs*. BD + EGb 1 h. n = 10 per group.

To address if the animal model of brain death induced kidney damage was successfully established and the effect of EGb761 on brain death-induced renal injury, we evaluated renal injury and function among groups. The results showed that BUN (Fig. 1A) and serum Cr (Fig. 1B) levels were significantly elevated in BD group than in sham group (both *P* < 0.01), suggesting renal injury in response to brain death. Compared to BD group, the BUN and serum Cr levels were significantly reduced in all EGb761-treated animals (all *P* < 0.01) except for the BD + EGb 2 h group. In addition, the timing of EGb761 injection seemed to be associated with the protective efficacy of EGb761. Among four EGb761-treated groups, the orders of BUN and serum Cr levels (from low to high) were BD + EGb b48h, BD + EGb 0.5 h, BD + EGb 1 h and BD + EGb 2 h, although the differences between groups did not always reach significance.

**Figure 2:**
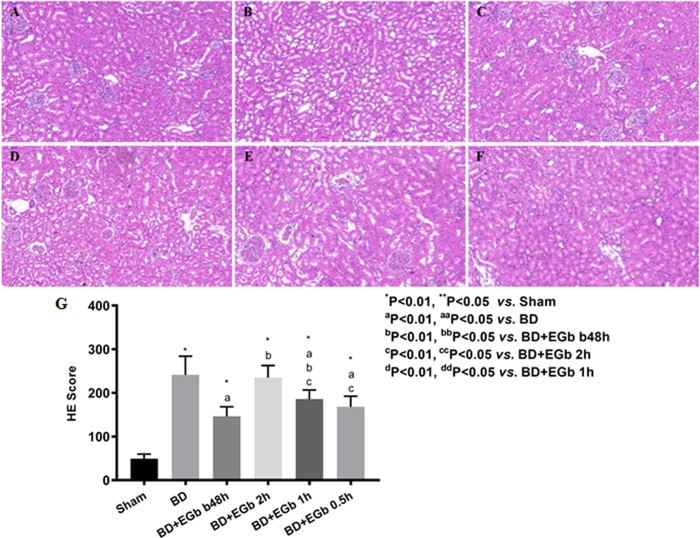
Histological analysis of kidney tissues from rats in the (**A**) sham, (**B**) BD, (**C**) BD + EGb b48h, (**D**) BD + EGb 2 h, (**E**) BD + EGb 1 h, and (**F**) BD + EGb 0.5 h groups. Tissue sections were H&E stained (200×). (**D**) The extent of kidney injury was quantified by histological scoring. Ten rats for each group, and ten fields from slide of each animal were evaluated.

Next, we compared the histological findings in kidney tissue among six groups. As shown in Fig. 2A, the image of the sham group exhibited an intact epithelial cell layer, and there was no tubule dilation, interstitial edema and infiltration of inflammatory cells. In addition, the brush border at the free side of the proximal convoluted tubule and longitudinal striation at the basement side was apparent. In contrast, the brain death group had dilated tubules containing granular casts, and apparent loss of tubular epithelial cells from the brush border, and some cells even had disintegrated (Fig. 2B). In the four EGb761-treated groups, the BD-induced injures were relieved in varying degrees as compared with BD group (Fig. 2C–F). The casts and brush border injury were also observed, but to a lesser extent than detected in the brain death group. The tubular injury score for six groups was shown in Fig. 2G. BD group had a highest injury score while sham group had a lowest one among six groups. Treatment with EGb761 significantly reduced the sore in brain death rats (except for BD + EGb 2 h group, all *P* < 0.01), and the order of scores and significances were consistent with those in the level of serum Cr.

**Figure 3:**
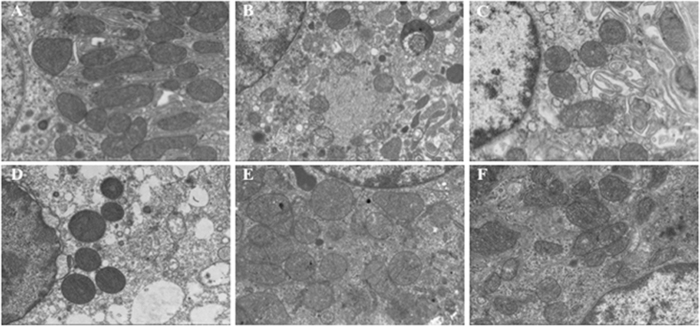
Representative electron microscopic examination of kidney tissues from rats in the sham (**A**), BD (**B**), BD + EGb b48h (**C**), BD + EGb 2 h (**D**), BD + EGb 1 h (**E**), and BD + EGb 0.5 h (**F**) groups.

The renal tissues were further evaluated with transmission electron microscopy. In sham group, the mitochondria exhibited intact structure, dark matrix and normal mitochondrial cristae. There was abundant endoplasmic reticulum with well-arranged structure (Fig. 3A). In BD group, the mitochondria showed swelled and had no intact structure, the matrix was blurred, and the structure of mitochondrial cristae was unclear. The endoplasmic reticulum was damaged and the structure became disorder (Fig. 3B). While in four EGb761-treated groups (Fig. 3C–F), the damages of mitochondria and endoplasmic reticulum were relieved in varying degrees as compared with BD group. In addition, the degree of recovery elevated with the increase of the duration of drug treatment.

Taken together, all these results suggested that the animal model of brain death-induced renal injury was successfully established, and EGb761 possessed protective effect against brain death-induced renal injury. Early administration of EGb761 can provide better protective efficacy.

### EGb761 downregulated the expression of pro-inflammatory cytokines and reduced macrophage infiltration

**Figure 4:**
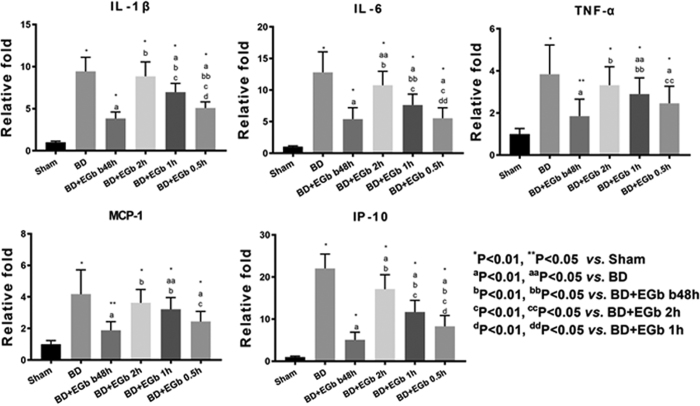
EGb761 reduced the levels of pro-inflammatory cytokines and chemokines in renal tissue of brain death rats. Real-time PCR was used to measure mRNA expression levels of IL-1β, IL-6, TNF-α, MCP-1 and IP-10 from renal tissue. The expression levels were normalized to GAPDH.

To investigate the possible mechanism underlying protective effect of EGb761 against brain death-induced renal injury, we determine the mRNA levels of inflammatory cytokines in renal tissue since EGb761 has been shown to possess good anti-inflammation properties^32^. As shown in Fig. 4, BD group had significantly higher expression of the pro-inflammatory cytokines IL-1β, IL-6, and TNF-α, and chemokines MCP-1 and IP-10 (*P* < 0.01) as compared with sham group. However, in four groups of EGb761-treated animals, the expression levels of IL-1β (except for BD + EGb 2 h, all *P* < 0.01), IL-6 (all *P* < 0.05), and TNF-α (except for BD + EGb 2 h, all *P* < 0.05), MCP-1 (except for BD + EGb 2 h, all *P* < 0.05) and IP-10 (all *P* < 0.01) significantly reduced as compared to BD group. In addition, the trends of reduced levels were consistently associated with the drug treatment time among all the five pro-inflammatory cytokines.

**Figure 5:**
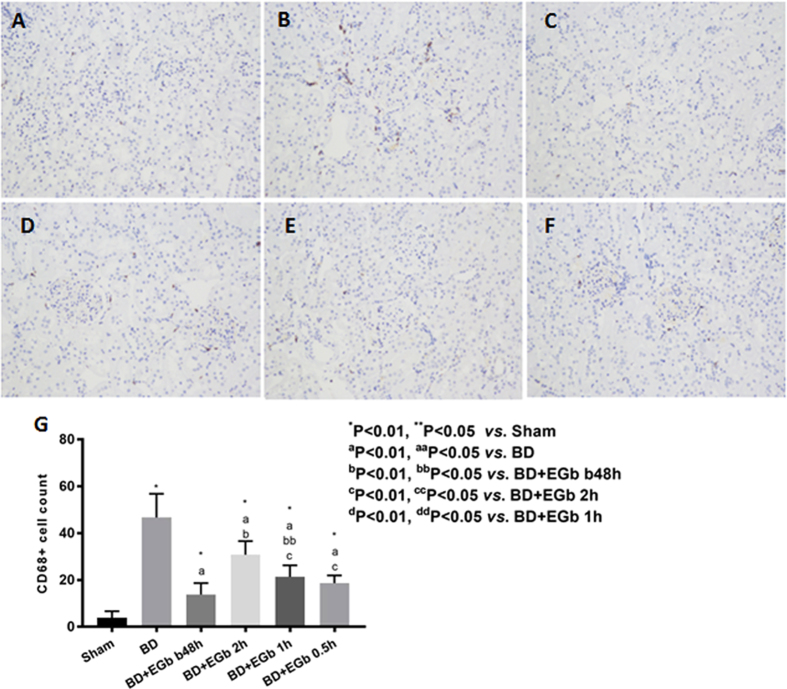
EGb761 reduced macrophage infiltration in brain death animals. IHC assessment was utilized to evaluate the extent of the CD68^+^macrophages infiltration in the kidneys of animals. Representative IHC staining images of CD68^+^ cells of sham (**A**), BD (**B**), BD + EGb b48h (**C**), BD + EGb 2 h (**D**), BD + EGb 1 h (**E**), and BD + EGb 0.5 h (**F**) groups were shown at a magnification of 400×. (**G**) The extent of macrophages infiltration was quantified by CD68^+^ cell count. Six rats for each group, and ten fields from slide of each animal were count.

To further confirm if macrophage infiltration in the kidney tissue was involved in the brain death-induced kidney injury, IHC assessment was performed to evaluate the extent of the CD68^+^ macrophages infiltration in the kidneys of animals (Fig. 5A–F). The CD68^+^ cell count significantly elevated in BD group as compared with sham group (*P* < 0.01, Fig. 5G). The extent of the macrophage infiltration significantly reduced in all four groups of EGb761-treated brain death animals as compared with BD groups (all *P* < 0.05), and the trends of reduced macrophage infiltration were consistent with those of the decreased levels of pro-inflammatory cytokines. Taken together, these data suggested that anti-inflammation properties of EGb761 may contribute to its protective effect.

### EGb761 inhibited SAPK and the JAK-STAT signalings

**Figure 6:**
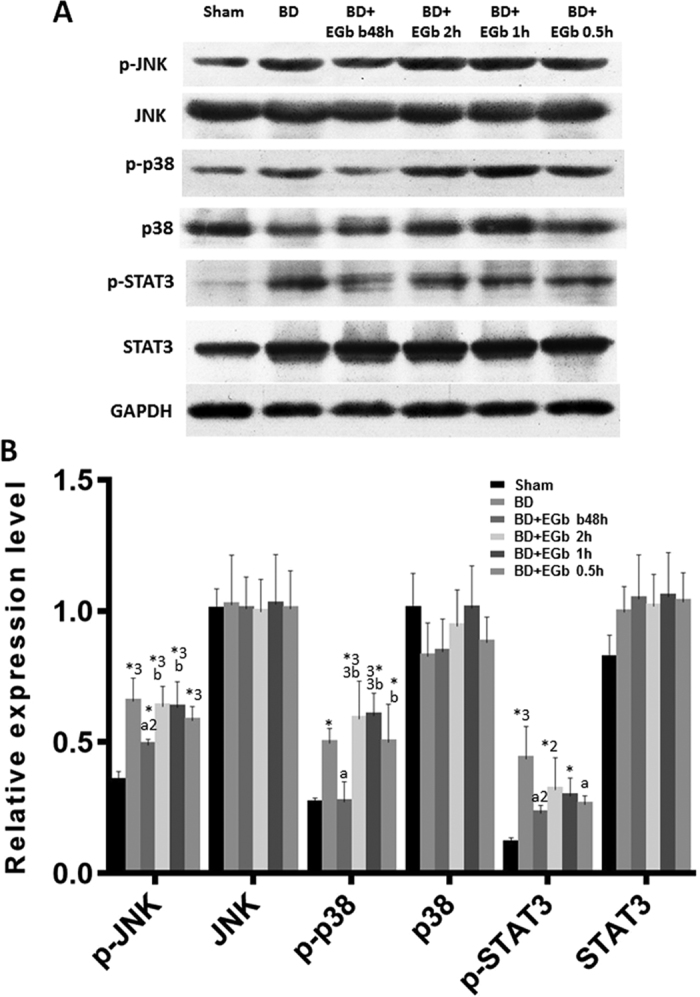
EGb761 inhibits SAPK and JAK-STAT signalings in the kidneys of brain death rats. (**A**) The expression and phosphorylation status of JNK-MAPK, p-38-MAPK, and STAT3 in renal tissue were assessed by Western blot. Representative immunoblots of the indicated proteins were shown. (**B**) Protein levels were quantified and normalized to GAPDH. Data was presented as mean ± SD. ^*^P < 0.05, ^*2^*P* < 0.01, ^*3^*P* < 0.001; significantly different from the sham group. ^a^*P* < 0.05, ^a2^*P* < 0.01 vs. BD; significantly different from the BD group. ^b^*P* < 0.05, ^b2^*P* < 0.01,^b3^*P* < 0.001; significantly different from the BD + EGb b48h group. n = 5 per group.

Next we further elucidated the molecular mechanism underlying protective effect of EGb761. Given the role of the JAK/STAT and SAPK pathways in inflammation and their connection to brain death induced peripheral organ damage, we assessed the phosphorylation state of key proteins in these intracellular signaling pathways. As shown in Fig. 6A,B, protein levels of p-JNK-MAPK (*P* < 0.001), p-p38-MAPK (*P* = 0.05), and p-STAT3 (*P* < 0.001) were significantly elevated in the BD group than in sham group. In addition, EGb761 treatment at 48 hours before brain death significantly reduced the levels of p-JNK-MAPK (*P* < 0.01), p-p38-MAPK (*P* < 0.05), and p-STAT3 (*P* < 0.01) as compared to BD group. However, in the three groups of BD rats receiving EGb761 after brain death, no significant differences was observed in p-JNK-MAPK (all *P* > 0.05), p-p38-MAPK (all *P* > 0.05), and p-STAT3 (all *P* > 0.05 except for BD + EGb 0.5 h group) as compared with BD group. There were no significant differences in the expression levels of total JNK-MAPK (*P* = 0.9996), p38-MAPK (*P* = 0.140), and STAT3 (*P* = 0.0749) among six groups (Fig. 6A,B). These data suggested that EGb761 treatment at 48 hours before brain death significantly inhibited SAPK and the JAK-STAT signalings, which may account for its protective effect.

## Discussion

In the current study, we evaluated the protective effect of EGb761 against brain death-induced kidney injury using a rat model of brain death. EGb761 treatment markedly reduced BUN and serum Cr level in the brain death rats. Both histological and transmission electron microscopy examinations demonstrated that EGb761 treatment significantly reduced brain death-induced injury in kidney tissues. Meanwhile, tubular injury score was also significantly reduced in EGb761-treated brain death rats. Comparing the data among four EGb761-treated demonstrated that the longer duration of drug treatment was, the better protective efficacy could be observed. In addition, RT-PCR data showed that EGb761 downregulated the expression of several pro-inflammatory cytokines, including IL-1β, IL-6, and TNF-α, MCP-1 and IP-10, as well as reduced macrophage infiltration in kidney tissue. Furthermore, western blot results showed that EGb761 treatment at 48 hours before brain death significantly inhibited SAPK and the JAK-STAT signaling in kidney tissues. Taken together, our findings suggested that EGb761 has the protective effect against brain death-induced kidney injury by inhibiting pro-inflammatory cytokines and the SAPK and JAK-STAT signalings. Early administration of EGb761 can provide better protective efficacy.

Brain death is a catastrophic event in the central nervous system, and is usually accompanied by extensive clinically significant changes in hemodynamics, systemic inflammation, endocrine disturbance, and severe renal injury. The combination of these processes markedly affects the structure and function of peripheral organs^10^. To better understand the mechanisms underlying this process, we established a progressive brain death model where the mean arterial pressure was maintained at or above 80 mmHg during the 4 h brain death. In the brain death animals, increased serum Cr and pathological tubular injury were observed, accompanied by increased pro-inflammatory cytokine (IL-1β, IL-6 and TNF-α) and chemokine (MCP-1 and IP-10) mRNA expression, suggesting the animal model was successful established and a clear role for inflammation in brain-death induced kidney damage.

The protective effect of EGb761 on renal I/R injury has been shown in previous studies. Sener *et al*. have reported that Ginkgo biloba extract EGb treatment ameliorates I/R-induced abnormalities, including alterations of several reactive oxygen metabolites biochemical indices, as well as histopathological morphology^29^. Likewise, Akdere *et al*. also demonstrated that EGb761 pretreatment significantly reduces tissue malondyaldehyde (MDA), catalase (CAT), and superoxide dismutase (SOD) levels and tissue damage in kidney tissue of I/R injured rats^25^. Although these findings suggest the protective potential of EGb761 on brain death-induced renal injury, however, its protective effect has not been investigated yet. To our best knowledge, this is the first study demonstrated the feasibility of application of EGb761 on pharmacologic donor management and the underlying molecular mechanism.

In previous studies, the effective dose of EGb761 for oral or intraperitoneal administration in different animal models generally range from 50 mg/kg to 200 mg/kg^33^,^34^,^35^,^36^. It has been shown that the *in vivo* bioavailability of oral administration of EGb761 is approximately 80%^37^, indicating that oral administration will result in a slightly lower serum level of EGb761 as compared with intravenous injection. In a rat model of I/R-induced renal injury by Sener *et al*., EGb761 exhibited its protective effect by intraperitoneal injection with a dose of 50 mg/kg twice before ischemia and reperfusion, respectively^29^. We chose intravenous administration with a dose of 50 mg/kg of EGb761 with the purposes to appropriately increase the *in vivo* bioavailability and to avoid the adverse effects caused by excessive drug dosage in animals.

Our data suggested that timing of administration is crucial for the protective efficacy of EGb761. In clinical practice, the time interval between confirmed diagnosis of brain death and donor organ procurement is typically longer than 6 hours, providing a good opportunity for using EGb761 to protect the donor kidney. Based on our results, earlier administration of EGb761 can achieve a better protection. The best protective efficacy was observed in the animals injected with EGb761 at 48 hours before brain death. EGb761 is a clinical used drug and possesses neuroprotective effect for patients with cerebrovascular disease^38^. For critical ill patients in the intensive care unit, EGb761 could be used to improve the patient’s condition, which could provide potential protection on the donor kidney before confirmed diagnosis of brain death. This is the rationale for our experimental design of BD + EGb b48h group. In addition, the rat model of brain death usually can be maintained up to 4–6 hours, which limits us to observe the long-term protective effect of EGb761. The BD + EGb b48h group can provide the data for evaluating the long-term protective effects of EGb761. Several intracellular signaling pathways have been identified to be involved in brain death-induced injury to peripheral organs. Bulcao *et al*. have demonstrated that the expression level of phosphorylated STAT3 increases up to five-fold in DBD grafts with donor heart dysfunction as compared to normal grafts^39^. Takahiro *et al*. showed that pretreatment of p38 MAPK inhibitor specifically inhibits p38-MAPK level in lung tissue and reduces serum pro-inflammatory cytokines in brain death animals^40^. Regarding brain death induced-renal injury, it has been shown that MAP-kinases-mediated inflammatory responses and NF-kappaB play a crucial role in the injury mechanism^41^. Previous study using pig model of brain death found that two major stress-activated protein kinases, JNKs and p-38 are involved in the brain death-induced kidney injury^42^. In addition, JNK and p38 has been shown to be involved in pathogenesis of several renal diseases, including acute renal failure, glomerulonephritis, and diabetic nephropathy through inducing inflammation and apoptosis^43^. In this study, we observed an elevated phosphorylation level of JNK-MAPK and p38-MAPK in the kidney tissue of brain death rats, suggesting that these signaling pathways were involved in the brain death-induced kidney damage. In addition, EGb761 pretreatment significantly reduced the phosphorylation levels, indicating that the protective effects of EGb761 against brain death-induced renal injury were, at least partially, mediated by inhibiting the SAPK signaling pathways.

The JAK-STAT signaling pathway is widely involved in sensing cellular stress, the inflammatory response, and immune regulation^44^. In patients with severe brain trauma and brain death, a large amount of IL-6 is released into the circulation^45^. Circulating IL-6 will binds to the gp130 receptor on cells, leading to the activation of the JAK-STAT signaling pathway^46^. In the current study, an increased level of phosphorylated STAT3, but not total STAT3 was observed in the kidney tissue of brain death rats, confirming that JAK-STAT signaling was activated. In addition, EGb761 treatment significantly inhibited JAK-STAT signaling, suggesting this signaling contributed to the protective effect of EGb761 agianst renal injury after brain death. Taken together, our findings suggested that SAPKs and the JAK-STAT pathways were involved in brain death-induced renal injury by evoking inflammatory responses, and these two signaling were involved in the molecular mechanism underlying protective effect of EGb761. Our results also suggested that strategy targeting to specific intracellular signaling pathways might have the potential to protect organs from brain death-induced damages.

There are still some limitations in this study. Firstly, although EGb761 attenuated brain death-induced kidney injury, the damages were not fully rescued as compared to sham group, indicating the donor management method of EGb761 pretreatment still has room for further improvement. Secondly, we did not further investigate the outcome of kidney transplantation from EGb761-treated DBD donors to comprehensively evaluate the protective effect of EGb761. Thirdly, although we found that EGb761 treatment inhibited SAPK and JAK-STAT signalings, we did not investigate the comprehensive molecular mechanism. In particular, previous studies have suggested that there are association between SAPK and JAK-STAT signaling^47^,^48^, which is worthy to be further investigated. All these limitations should be considered in the following study.

In conclusion, our findings demonstrated that EGb761 can protect renal from brain death-induced damage by inhibiting the inflammatory response and SAPK and JAK-STAT signaling in rats. Early administration of EGb761 can provide better protective efficacy, which may improve the outcome of kidney transplantation. Further studies are required to validate these findings.

## Additional Information

**How to cite this article:** Li, Y. *et al*. Ginkgo biloba extract EGb761 attenuates brain death-induced renal injury by inhibiting pro-inflammatory cytokines and the SAPK and JAK-STAT signalings. *Sci. Rep.* **7**, 45192; doi: 10.1038/srep45192 (2017).

**Publisher's note:** Springer Nature remains neutral with regard to jurisdictional claims in published maps and institutional affiliations.
